# Electroacupuncture improves gout arthritis pain via attenuating ROS-mediated NLRP3 inflammasome overactivation

**DOI:** 10.1186/s13020-023-00800-1

**Published:** 2023-07-18

**Authors:** Huina Wei, Boyu Liu, Chengyu Yin, Danyi Zeng, Huimin Nie, Yuanyuan Li, Yan Tai, Xiaofen He, Boyi Liu

**Affiliations:** 1 Department of Neurobiology and Acupuncture Research, The Third Clinical Medical College, Key Laboratory of Acupuncture and Neurology of Zhejiang Province, Zhejiang Chinese Medical University, Hangzhou, 310053 China; 2 Academy of Chinese Medical Sciences, Zhejiang Chinese Medical University, Hangzhou, 310053 China

**Keywords:** Inflammasome, Inflammation, Pain, Gout, TRPV1, Acupuncture

## Abstract

**Background:**

Gout results from disturbed uric acid metabolism, which causes urate crystal deposition in joints and surrounding tissues. Gout pain management is largely limited to colchicine and nonsteroidal anti-inflammatory drugs. Constant usage of these medications leads to severe side effects. We previously showed electroacupuncture (EA) is effective for relieving pain in animal model of gout arthritis. Here we continued to study the mechanisms underlying how EA alleviates gout pain.

**Methods:**

Monosodium urate was injected into ankle joint to establish gout arthritis model in mice. EA or sham EA was applied at ST36 and BL60 acupoints of model animals. Biochemical assays, immunostaining, live cell Ca^2+^ imaging and behavioral assays were applied.

**Results:**

Model mice displayed obvious mechanical allodynia, accompanied with gait impairments. EA attenuated mechanical hypersensitivities and improved gait impairments. EA reduced the overexpression of NLRP3 inflammasome signaling molecules in ankle joints of model animals. EA-induced anti-allodynia, as well as inhibition on NLRP3 inflammasome, were mimicked by antagonizing but abolished by activating NLRP3 inflammasome via pharmacological methods. EA attenuated oxidative stress, an upstream signaling of NLRP3 inflammasome in ankle joints of model mice. Exogenously increasing oxidative stress abolished EA’s inhibitory effect on NLRP3 inflammasome and further reversed EA’s anti-allodynic effect. EA reduced neutrophil infiltrations in ankle joint synovium, a major mechanism contributing to oxidative stress in gout. Pharmacological blocking NLRP3 inflammasome or EA reduced TRPV1 channel overexpression in dorsal root ganglion (DRG) neurons. Ca^2+^ imaging confirmed that EA could reduce functional enhancement in TRPV1 channel in DRG neurons during gout.

**Conclusions:**

Our results demonstrate that EA reduces gout pain possibly through suppressing ROS-mediated NLRP3 inflammasome activation in inflamed ankle joints and TRPV1 upregulation in sensory neurons, supporting EA as a treatment option for gout pain.

**Supplementary Information:**

The online version contains supplementary material available at 10.1186/s13020-023-00800-1.

## Introduction

Gout arthritis results from disturbed uric acid metabolism, which causes monosodium urate (MSU) crystal deposition in joints [1]. It is a very common inflammatory arthritis condition in the world [1, 2]. It triggers joint inflammation and causes severe pain [3]. The severe pain further affects the gait of suffering patients, which in together affects their daily activities and life qualities. Unfortunately, the occurrence rate of gout arthritis is steadily increasing because of the aging populations and changes in people’s eating habits [1]. The patients are usually prescribed with NSAIDs, colchicine or even corticosteroids for gout pain management [4]. But these medications are known to cause obvious and severe side effects if used constantly [4]. Thus, identifying alternative approaches with minimal toxic side effects to alleviate gout arthritis pain has clinical importance.

A potential treatment approach that met such criteria is acupuncture. Acupuncture has been used for pain relief since ancient times. Recently, some meta-analyses concluded that acupuncture is an effective alternative therapy for gouty arthritis patients to control pain and improve their life qualities [5, 6]. In animal studies, our recent work found that electroacupuncture (EA) could relieve mechanical allodynia in a rat model of gout arthritis [7]. Although the therapeutic potentials of acupuncture on gout arthritis pain in patients has been documented and summarized, it still remains elusive how exactly acupuncture may exert its beneficial effects on gout arthritis at this stage.

MSU deposition in the joint can activate the innate immune response, including neutrophil and macrophage influxes that produce inflammations [8]. MSU triggers activation of NLRP3 inflammasome when engulfed by infiltrated macrophages [9]. NLRP3 inflammasome then cleaves pro-IL-1β to generate mature IL-1β, which produces pro-inflammatory effect and contributes to gout pain by either directly activating or sensitizing peripheral nociceptors, including upregulating the pain-sensing transient receptor potential cation channel subfamily V member 1 (TRPV1) channel [10]. The research from us and from other peers in together demonstrated that oxidative stress occurred in local inflamed joint during gout as a result of neutrophil infiltration, which produces excessive ROS and contributes to gout pain [11–13]. Moreover, ROS also acts as an important contributor to NLRP3 inflammasome activation during gout [12, 14]. These findings in together suggest ROS-mediated NLRP3 inflammasome activation as an important mechanism underlying gout arthritis pain.

Here, we established a mouse model for gout arthritis and studied the effect of EA on mechanical allodynia as well as gait impairment. We showed that EA reduces ROS-mediated NLRP3 inflammasome over-activation in ankle joint and TRPV1 up-regulation in sensory neurons, all of which may contribute to EA’s interventional effects on the animal model of gout arthritis pain.

## Methods and materials

### Animals

C57BL/6J mice (male, around 8 weeks, 20–25 g) were purchased in Shanghai Laboratory Animal Center. Ly6G-IRES-GFP knock in mouse line were generated via Cas9-associated guide RNA (gRNA) technique by Shanghai Model Organisms Center, Inc. (Shanghai, China). Animals were fed under standard environmental conditions (12 h light–dark cycle, temperature: 22–24 °C, humidity: 40–60%). Five mice were housed in one cage.

### Mouse gout arthritis model establishment

The gout arthritis model was induced by intra-articular (i.a.) injection of MSU crystals (500 μg/20 μl, Sigma-Aldrich, USA) into ankle joint of mice under isoflurane anesthesia as described before [15]. Endotoxin-free PBS was used to prepare MSU crystal suspension. The control group of mice received an i.a. injection of 20 μl sterile PBS. Ankle oedema and mechanical hypersensitivity which occurred 2 h after injection was regarded as a criterion for successful establishment of the model as reported before [11].

### Ankle joint mechanical allodynia and heat hyperalgesia assessment

Mice were habituated to the testing environment for 3 days prior to formal test. Mice were put in transparent Plexiglas chambers on an elevated mesh floor. The animals were accustomed to the test environment for at least 30 min before start. The mechanical allodynia was tested by von Frey filament by “Up-Down” method as previously described [16, 17]. 50% paw withdrawal thresholds (PWTs) were deduced through Dixon test [18]. Mechanical allodynia was measured at specific time points as shown in the figures. Heat hyperalgesia was evaluated via by Hargreaves test [19]. In brief, a radiant light beam generated by a light bulb was directed to the right hind paw to measure paw withdrawal latency (PWL). A cutoff threshold of 20 s was set to avoid tissue injury. Behavior tests were all conducted by an experimenter blinded to experimental conditions.

### Gait assessment

Gait of mice was monitored and then analyzed via DigiGait^™^ system (MouseSpecifics, Inc., USA) as previously described [20]. Briefly, the mouse was put on a flat and transparent treadmill which was operated on a constant speed (18 cm/s). A video camera was located underneath the treadmill to record the gait of the mouse while it was running. The animals ran on the treadmill for a period of 20 s and a consecutive of five strides were averaged per animal and used for analysis. Parameters including paw area, swing and stride length were calculated through the software and then summarized and compared.

### Electroacupuncture (EA) intervention

The mice were immobilized with a restrainer. Acupuncture needles (0.16 mm diameter) were inserted at a depth of 4 mm into bilateral Zusanli (ST36, 5 mm lateral to the anterior tubercula of the tibia) and Kunlun (BL60, at the ankle joint level and between the tip of the external malleolus and calcaneus) acupoints [21, 22]. Sham EA-treated mice received shallow needle insertion but with no electric stimulation [21, 23]. All other groups of mice received the same immobilizing treatment as EA or sham EA group. The needles were connected with HANS-200A acupuncture point nerve stimulator. Interventional parameters: 2/100 Hz alternating frequency, 0.5 mA stimulating intensity, 30 min intervention/session. The EA/sham EA interventions were carried out at 7.5 h and 23.5 h time points, respectively.

### Drug administration

MCC950 (APExBIO Technology, TX, USA, 20 mg/kg) was prepared in stock solution in DMSO and further diluted in PBS (1:33) prior to use, and was applied via intraperitoneal (i.p.) rout. The dose was based upon former study [24]. Vehicle containing (3.0% DMSO in PBS) was used as control. The above drug treatments were administered at 6 and 22 h time points after MSU injection. Nigericin (Selleckchem, USA), an activator of NLRP3, was dissolved in DMSO and was further diluted in PBS (1:100) and injected into the joint of mice at a dose of 100 μg/20 μl/site (i.a.) 0.5 h ahead of EA intervention. The control group mice received 1% DMSO injection. H_2_O_2_ was diluted to 0.3% in PBS and injected into ankle joint of mice at a dose of 0.3%/40 μl/site. The control group mice received PBS injection.

### Determination of oxidant/antioxidant status and ROS products

The detailed protocols were documented in our previous publication [12]. Briefly, the ankle tissue was taken 24 h after model establishment, and used for oxidative stress biomarkers examination. All samples are chopped and centrifuged and then the supernatant was collected for corresponding biochemical assays. The collected supernatants were then analyzed by means of commercially available kits from Nanjing Jiancheng Bioengineering Institute (China) and Beyotime Biotechnology (China).

### Determination of the hind paw swelling

Ankle swelling was observed as an increase in hind paw diameter, as measured by a digital caliper, and was calculated as the difference between the basal value and the test value as in our previous study [11]. Changes in ankle diameter were shown as % increase in diameter and calculated as follows: % increase in ankle diameter = *D*_after_/*D*_before_◊100%. Each mice was measured 3 times, and the mean value was calculated.

### Histopathological assessment of ankle joint

Mice were euthanized 24 h after MSU injection. Ankle joints were fixed with 10% paraformaldehyde in PBS, and then decalcified for 30 d with EDTA and embedded in paraffin for histological analysis. The paraffin sections were stained with hematoxylin and eosin for conventional morphological evaluation under a light microscope (DM 2500, Leica, Germany) with 10 × and 40 × objectives. The numbers of infiltrated inflammatory cells per observation field in different groups were counted in a blind manner using 40 × objectives.

### Immunofluorescent staining

Detailed procedures were described in our previous publication [25]. Briefly, the mice were deeply anesthetized with isoflurane, and were perfused through ascending aorta with 0.9% saline followed by 4% paraformaldehyde in 0.01 M PBS. After perfusion, the ipsilateral L3‐5 DRGs were removed and post‐fixed in the same fixative for 4–6 h (4 °C) before transferring to 15% and 30% sucrose for 72 h for dehydration. Longitudinal DRG Sects. (8 µm) were cut on a frozen microtome (Thermo NX50, MA, USA), and then processed for immunofluorescence. The tissues were first blocked with 5% donkey serum in Tris buffered saline tween (TBST) for 1 h at 37 °C, then they were incubated overnight with the corresponding primary antibody at 4 °C. The primary antibodies used were rabbit anti-TRPV1 (1:1,000, #ACC‐030, Alomone Labs), mouse anti NeuN (1:400, #ab104244, Abcam). After washing, the tissues were incubated with corresponding secondary antibodies (1:1,000, #ab150064&#ab150061, Abcam) for 2 h at 37 °C. Images were captured by Zeiss Axio Imager M2 (Zeiss, Germany) microscope. The number of TRPV1 positively stained DRG neurons were divided by the total number of DRG neurons identified by positive NeuN staining in order to calculate % of TRPV1 positive neurons. Three images were randomly selected from each tissue section, averaged, and then analyzed according to methods we previously described [26, 27].

### Western blotting

To measure the protein expressions of NLRP3, Caspase-1, ASC, IL-1β, IL-18 and 4-HNE, ankle joint samples were harvested at 24 h time point. Samples were homogenized in RIPA buffer [50 mM Tris (pH 7.4), 150 mM NaCl, 1% Triton X-100, 1% sodium deoxycholate, sodium orthovanadate, 0.1% SDS, EDTA, sodium fluoride, leupeptin, and 1 nM PMSF], then centrifuged at 12,000 rpm for 12 min at 4 °C and the supernatant was collected. The protein concentration was determined using BCA method according to the kit’s instruction (Thermo Fisher, USA) and 30 μg protein was loaded in each lane. Protein was loaded and separated by SDS‐PAGE and electrophoretically transferred onto PVDF membranes. The membranes were blocked with 5% non-fat milk in TBST solution for 1 h at room temperature, and then the membranes were incubated with primary antibodies overnight at 4 °C as we previously described [28, 29]. The antibodies used are as follows and further diluted in TBST: IL-1β (1:800, # ab9722, Abcam), ASC (1:500, rabbit polyclonal, #PA5-88132, Thermo Fisher); IL-18 (1:500, rabbit polyclonal, #ab191860, Abcam), NLRP3 (1:800, #NBP2-12446, Novus), Caspase-1 (1:1000, #24232s, CST) and 4-HNE (1:500, #ab62623, Abcam). Subsequently, the immunoblots were incubated with the secondary antibodies for 2 h at room temperature. β- actin (1:5000, #ab20272, Abcam) was used as the reference control. The mean expression level of the target protein in the control group was considered as 100%, and the relative expression level of the target protein in all other groups was adjusted as a ratio to the level of the control group.

### Ca^2+^ imaging

Ipsilateral L3-L5 dorsal root ganglion (DRG) were dissociated one day after MSU injection. Details for the digestion of DRG, neuron culture and ratiometric Ca^2+^ dye Fura-2 incubation were documented in details in our recent publications [25, 30]. The DRG neurons were used for Ca^2+^ imaging within 4 h after culture. A cell was considered a positively responding cell in this study when the Ca^2+^ response reached over 10% of its baseline.

### Statistical analysis

The GraphPad Prism 8 was used for all data analyses in this study. Data in bar graphs are expressed as means ± SEM. One- or two-way ANOVA with repeated measures followed by Tukey’s *post-hoc* test was used for comparison among groups. Student’s *t*-test was used for comparisons between 2 groups. Comparison is deemed as significantly different if *p* < 0.05.

## Results

1. EA intervention attenuates mechanical allodynia and improves gait impairment in gout arthritis model mice.

**Fig. 1:**
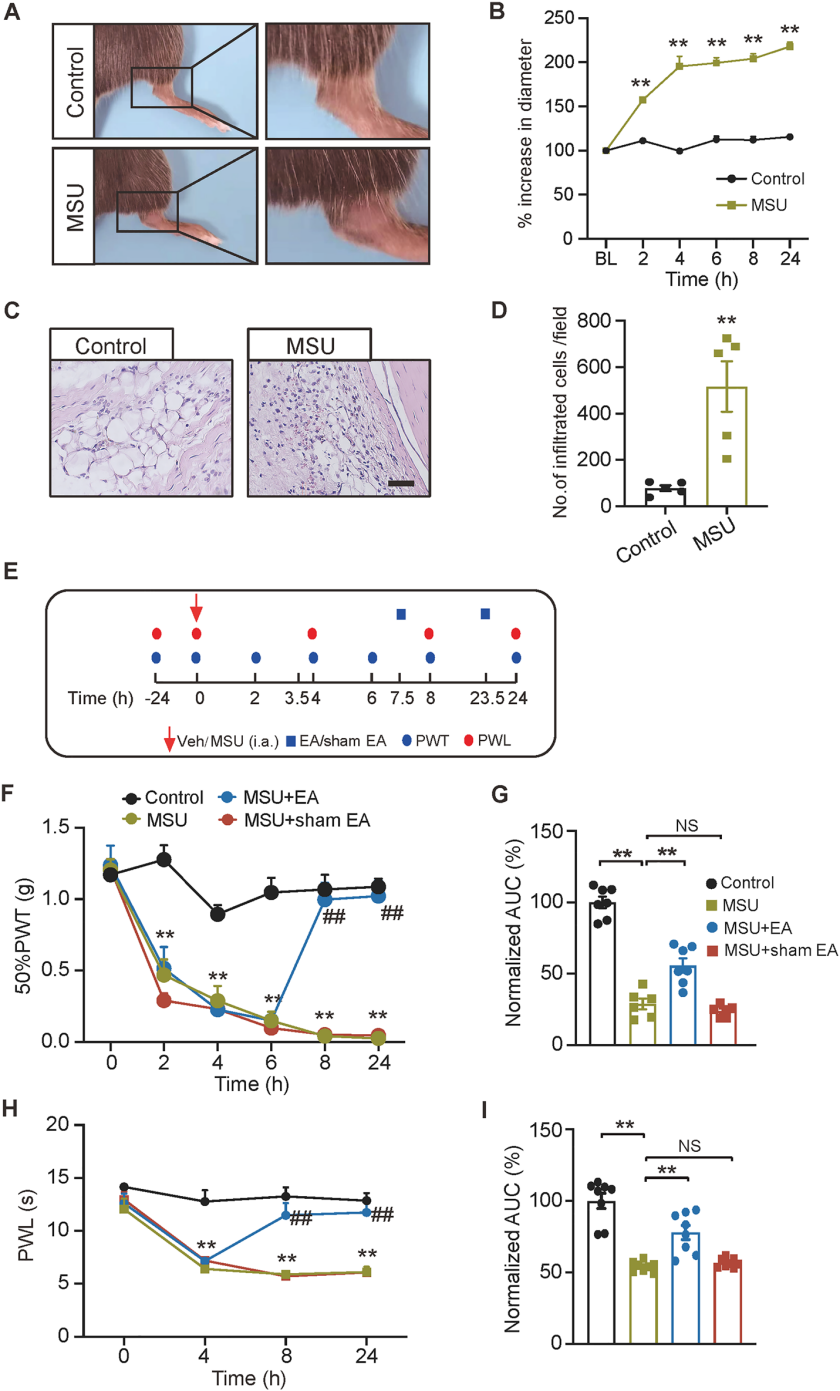
The establishment of the gout arthritis mouse model and EA’s intervention on pain in mice. **A** Representative photos showing ipsilateral ankles injected with sterile PBS (control group) or MSU (+ MSU group). The right panels denote the enlarged ankle joint. **B** Time courses showing the percent increase in ankle diameter of control and MSU group mice. BL: basal level. **p < 0.01 vs. control group. **C** Representative photos of pathological sections of ankle joint synovium from control and MSU group mice. The scale bar indicates 20 μm. **D** Summary of the number of infiltrated cells per observation field. **p < 0.01 vs. control group. **E** Experimental protocol illustrating time points for model establishment, EA/sham EA interventions and mechanical/thermal pain measurements. **F** 50% PWT changes of control, MSU, MSU + EA and MSU + sham EA groups. **p < 0.01 vs. control group. ^##^*p* < 0.01 vs. MSU + sham EA. **G** Normalized AUC analysis of curves shown in panel F. The value from the control group was taken as 100%, and other groups were normalized thereafter. **H** Time course showing PWL changes of 4 groups. **p < 0.01 vs. control group. ^##^*p* < 0.01 vs. MSU + sham EA. **I** Normalized AUC analysis of the data in panel H. n = 5–8 mice/group. **p < 0.01, *NS* no significance

The right ankles of the mice were injected with MSU intra-articularly (i.a.) to establish the gout arthritis model [15]. Control group received only sterile PBS injection. MSU injection elicited obvious ankle edema, which lasted over 24 h, compared with control group (Fig. 1A, B, MSU vs. control). Ankle pathology revealed that MSU injection triggered robust inflammatory cell infiltration into synovium of ankle joints compared with control group (Fig. 1C, D). MSU injection further produced obvious mechanical allodynia in ipsilateral hindpaw, which lasts > 24 h. These observations were all similar with results of former studies, demonstrating the successful establishment of the model [11, 15].

We then evaluated the interventional effect of EA on mechanical pain of gout model mice. From our previous study, it was found 2/100 Hz EA as an effective intervention frequency to control mechanical pain in an animal model of gout arthritis [7]. Here, we continued to apply EA with 2/100 Hz frequency on bilateral ST36 and BL60 acupoints of gout model mice. EA was applied at 7.5 and 23.5 h after model establishment as indicated (Fig. 1E). We found EA’s intervention obviously alleviated mechanical pain of gout model mice (Fig. 1F, MSU + EA vs. MSU). Area under the curve (AUC) analysis indicated the overall anti-allodynia effect across the evaluated time points. In contrast, sham EA did not show such effect (Fig. 1G, MSU + sham EA vs. MSU). Furthermore, gout model mice developed obvious heat hyperalgesia, manifested by the reduction in PWT upon noxious heat stimuli compared with control group. The heat hyperalgesia of gout model mice was significantly alleviated by EA (Fig. 1H, I, MSU + EA vs. MSU). In contrast, sham-EA group did not exhibit such improvement (Fig. 1H, I, MSU + sham EA vs. MSU).

**Fig. 2:**
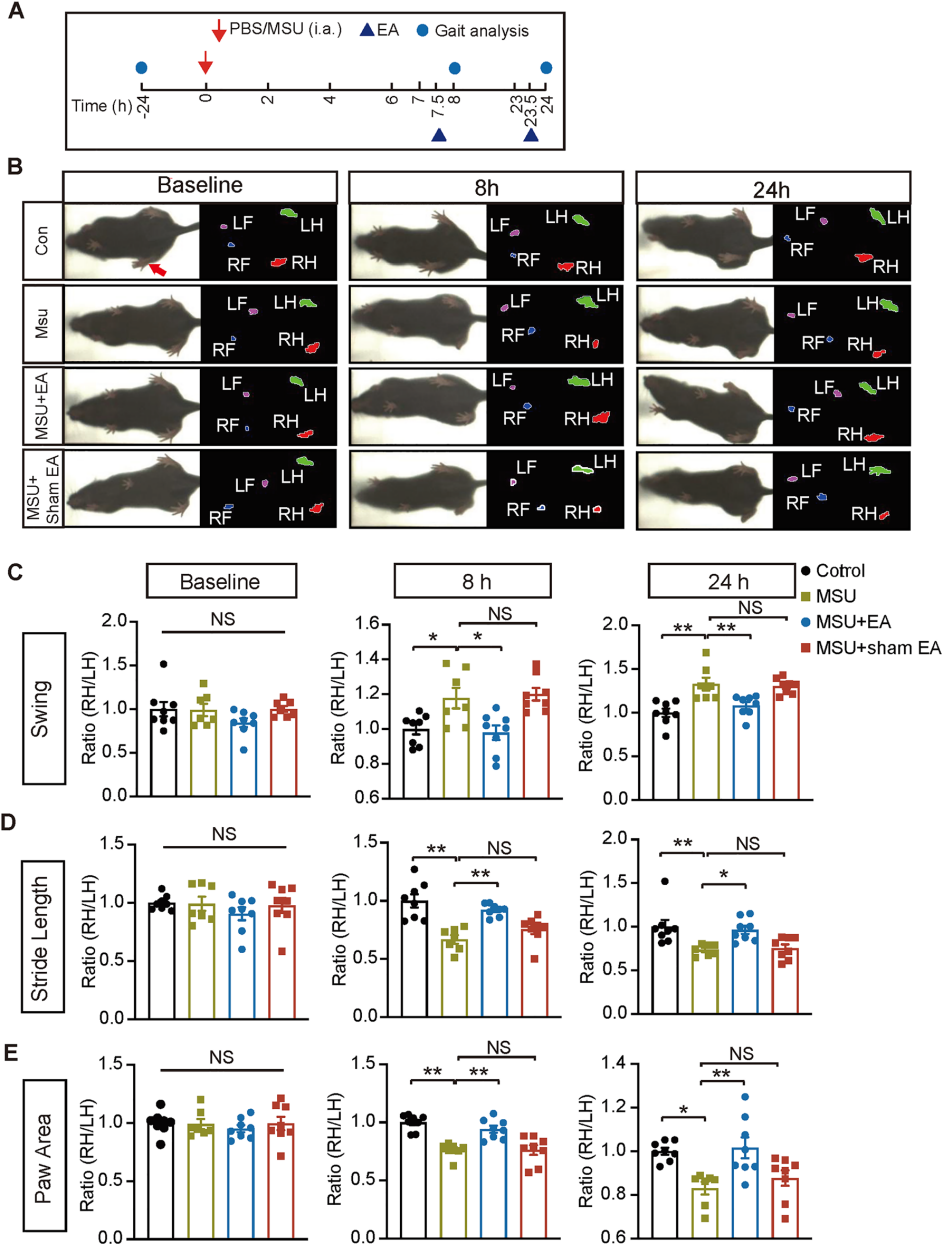
EA intervention improved gait impairments of gout arthritis model mice. **A** Schedule for model establishment, gait analysis and EA/sham EA treatment. **B** Representative pictures illustrating the mice from each group recorded by the gait analysis system at 0 (baseline), 8 or 24 h time points after model establishment. The left panel shows the instant images of the mice on the treadmill. The right panel shows the paw area being analyzed. The letter L or R denote left or right, whereas F or H denote forepaw or hind paw, respectively. **C**–**E** Summary of paw area ratio (RH/LH), stride length ratio (RH/LH) and swing ratio (RH/LH) of baseline (− 24 h), 8 h and 24 h time points. n = 7–8 mice/group. *p < 0.05, **p < 0.01. *NS* no significance

It is known that gout pain triggers gait impairment among patients, which severely restricts mobility of the lower limbs and impacts daily activities [31]. We explored whether gout arthritis model mice might develop similar gait impairment as gouty patients and if so, whether EA intervention could reverse it. Animal’s gait was recorded via DigiGait analyzing system at 8 and 24 h time point after model establishment (Fig. 2A). Gait analysis showed that all 4 groups of mice exhibit similar gait before model establishment [Fig. 2B and C–E left panels, baseline (− 24 h)]. However, MSU group mice exhibited significant gait impairments 8 and 24 h after model establishment vs. control group. Specific gait parameters, including swing ratio, stride length ratio and paw area ratio were all significantly impaired in MSU group at 8 and 24 h time points compared with control group (Fig. 2C–E middle and right panels, MSU vs. control). EA intervention significantly improved these impaired gait parameters at both 8 and 24 h time points in gout model mice (Fig. 2C–E middle and right panels, MSU + EA vs. MSU). In contrast, sham EA had no such effect (Fig. 2C–E middle & right panels, MSU + sham EA vs. MSU). We further examined the effect of EA on gait impairment with detailed spatiotemporal characterization of the footprints. The analysis revealed that MSU-injected mice developed abnormal dynamic changes in the area of the right (ipsilateral) hind paw compared to the left (contralateral) hind paw at both 8 and 24 h time points (Additional file 1: Fig. S1A). EA intervention reversed the impaired dynamic changes in paw area of the ipsilateral hind paw (Additional file 1: Fig. S1A). We further summarized and overlaid the time courses showing dynamic changes in paw area of the right hind paw from all group of mice at 8 or 24 h time point (Additional file 1: Fig. S1B, C). MSU group showed significantly impaired ensemble area of right hind paw vs. control group at these two time points, whereas EA intervention significantly improved the impairments (Additional file 1: Fig. S1B, C, MSU + EA vs. MSU). But sham EA had no such effect (Additional file 1: Fig. S1B, C, MSU + sham EA vs. MSU). Therefore, these results demonstrate that EA intervention significantly ameliorates mechanical pain and improves gait impairments in gout arthritis model mice.

2. The intervention of EA reduced NLRP3 inflammasome activation and pro-inflammatory cytokine overproduction of inflamed ankle joints of gout arthritis model mice.

**Fig. 3:**
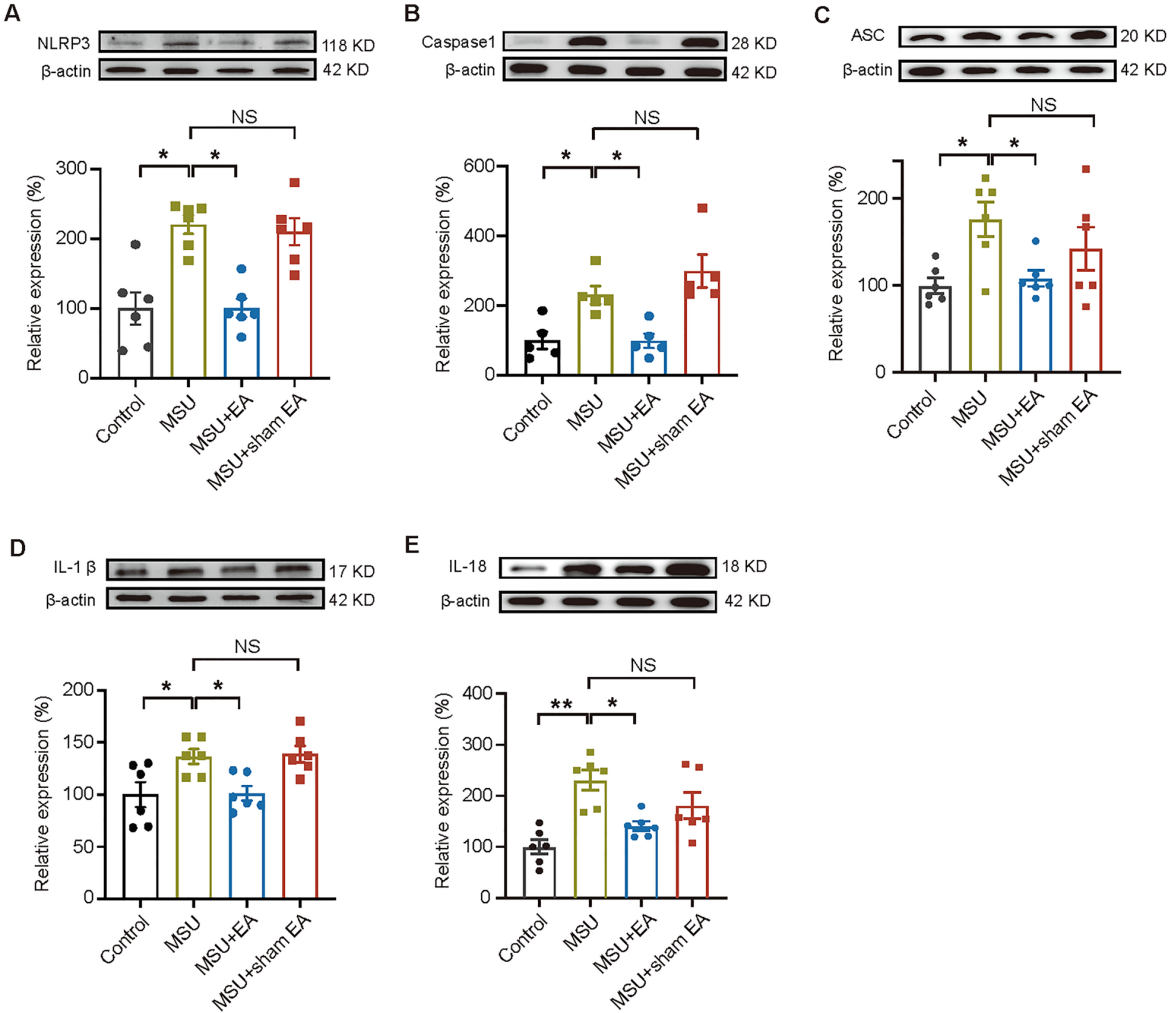
EA intervention attenuated NLRP3 inflammasome activation in ankle joints of gout arthritis model mice. **A**–**E** Western blotting examination of protein expressions of NLRP3 inflammasome signaling components, including NLRP3 (**A**), Caspase-1 (**B**) ASC (**C**) and inflammatory cytokines IL-1β (**D**), IL-18 (**E**) in ankle joint tissues from control, MSU, MSU + EA and MSU + sham EA groups of mice. Upper panels showed the representative Western blot gels, whereas lower panels indicated the summarized data. n = 5–6 mice/group. *p < 0.05. *NS* no significance

NLRP3 inflammasome consists of a complex of proteins responsible for cleaving pro-IL-1β and pro-IL-18 into active IL-1β and IL-18. It is known that IL-1β is a critical inflammatory cytokine involved in gout pain and inflammation [32]. We thus planned to explore if EA might be able to interfere with NLRP3 inflammasome signaling. Western blot indicated that protein expressions of NLRP3, Caspase-1, ASC as well as inflammatory cytokines IL-1β, IL-18 were obviously elevated in the inflamed ankle joints of MSU group *vs*. control group (Fig. 3A–E, MSU vs. control). EA remarkably reduced the elevated protein expressions of NLRP3, Caspase-1, ASC, IL-1β and IL-18 in ankle joint tissues (Fig. 3A–E, MSU + EA vs. MSU). In contrast, sham EA exhibited no any such effects (Fig. 3A–E, MSU + sham EA vs. MSU). These results demonstrate that EA can reduce NLRP3 inflammasome activation in the inflamed ankle joints of gout model mice.

3. The analgesic effects of EA are mimicked by antagonizing NLRP3 inflammasome and reversed by activating NLRP3 inflammasome.

**Fig. 4:**
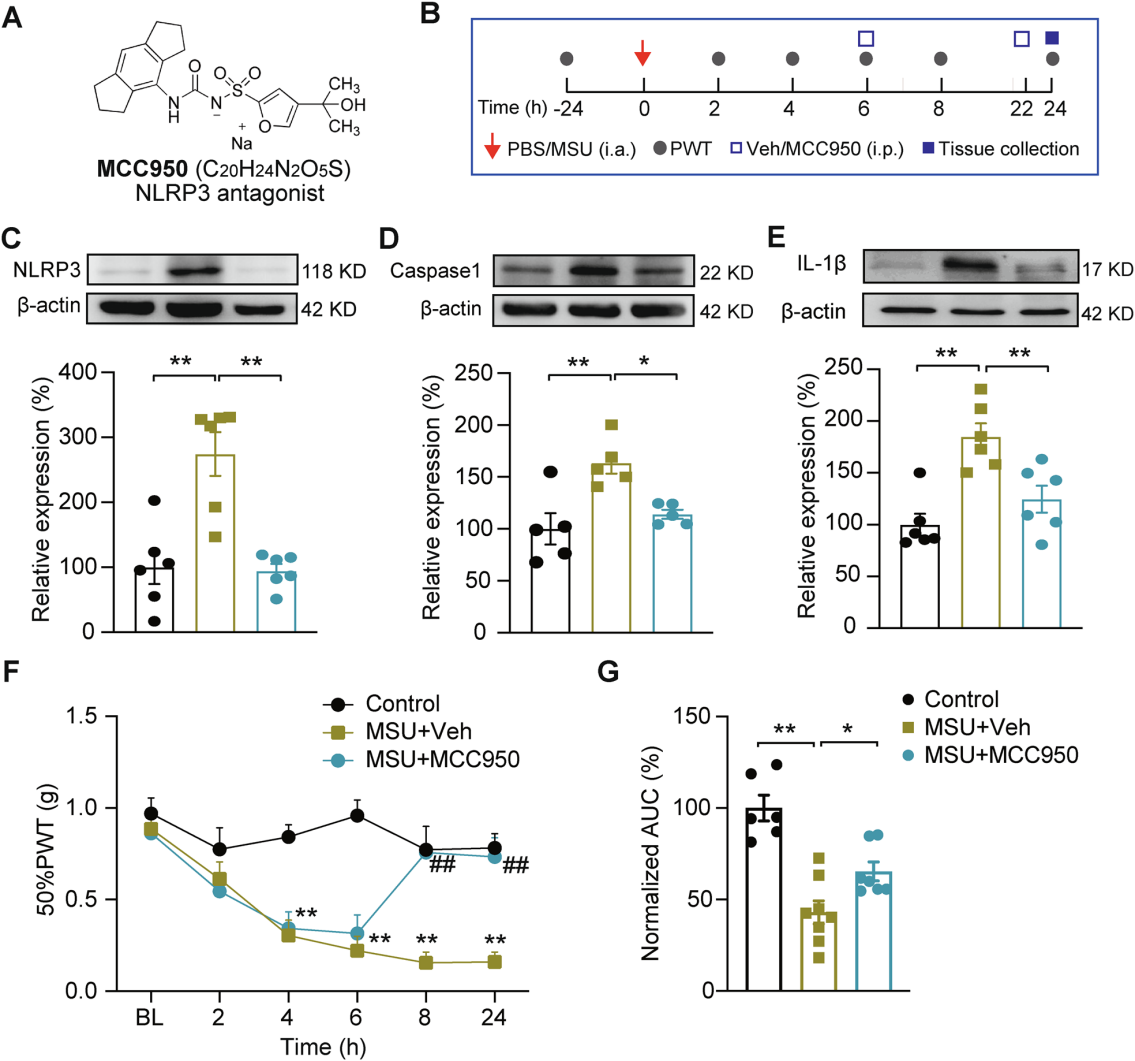
Specific blocking NLRP3 inflammasome activation ameliorates mechanical allodynia of gout arthritis model mice. **A** Molecular structure of MCC950. **B** Schedual illustrating model establishment, MCC950 administration, PWT test and tissue collection. **C**–**E** Western blotting examination of protein expression of NLRP3 inflammasome components, including NLRP3 (**C**), Caspase-1 (**D**) and IL-1β (**E**) in ankle joints from control, MSU + Veh and MSU + MCC950 groups of mice. *p < 0.05, **p < 0.01. **F** Time courses showing 50% PWT changes of ipsilateral hindpaws. **p < 0.01 vs. control group. ^##^*p* < 0.01 vs. MSU + Veh group. **G** Normalized AUC analysis of the data in panel F. *p < 0.05, **p < 0.01. n = 5–7 mice/group

MCC950 was identified as a specific blocker for NLRP3 inflammasome activation [10, 24] (Fig. 4A). We proceeded to investigate whether pharmacological blocking NLRP3 inflammasome activation by MCC 950 could result in similar anti-allodynic effect as EA. MCC950 was administered intraperitoneally (20 mg/kg, i.p.) at 6 and 22 h time points after establishing the model (Fig. 4B). MCC950 treatment significantly alleviated NLRP3, Caspase-1 and IL-1β protein overexpression in ankle joint tissues of gout model mice compared with vehicle-treated (control) group (Fig. 4C–E), confirming the effectiveness of MCC950 on NLRP3 inflammasome. Furthermore, MCC950 treatment significantly ameliorated mechanical allodynia of gout model mice (Fig. 4F, G), demonstrating the critical contribution of NLRP3 inflammasome to mechanical pain of gout arthritis model mice.

**Fig. 5:**
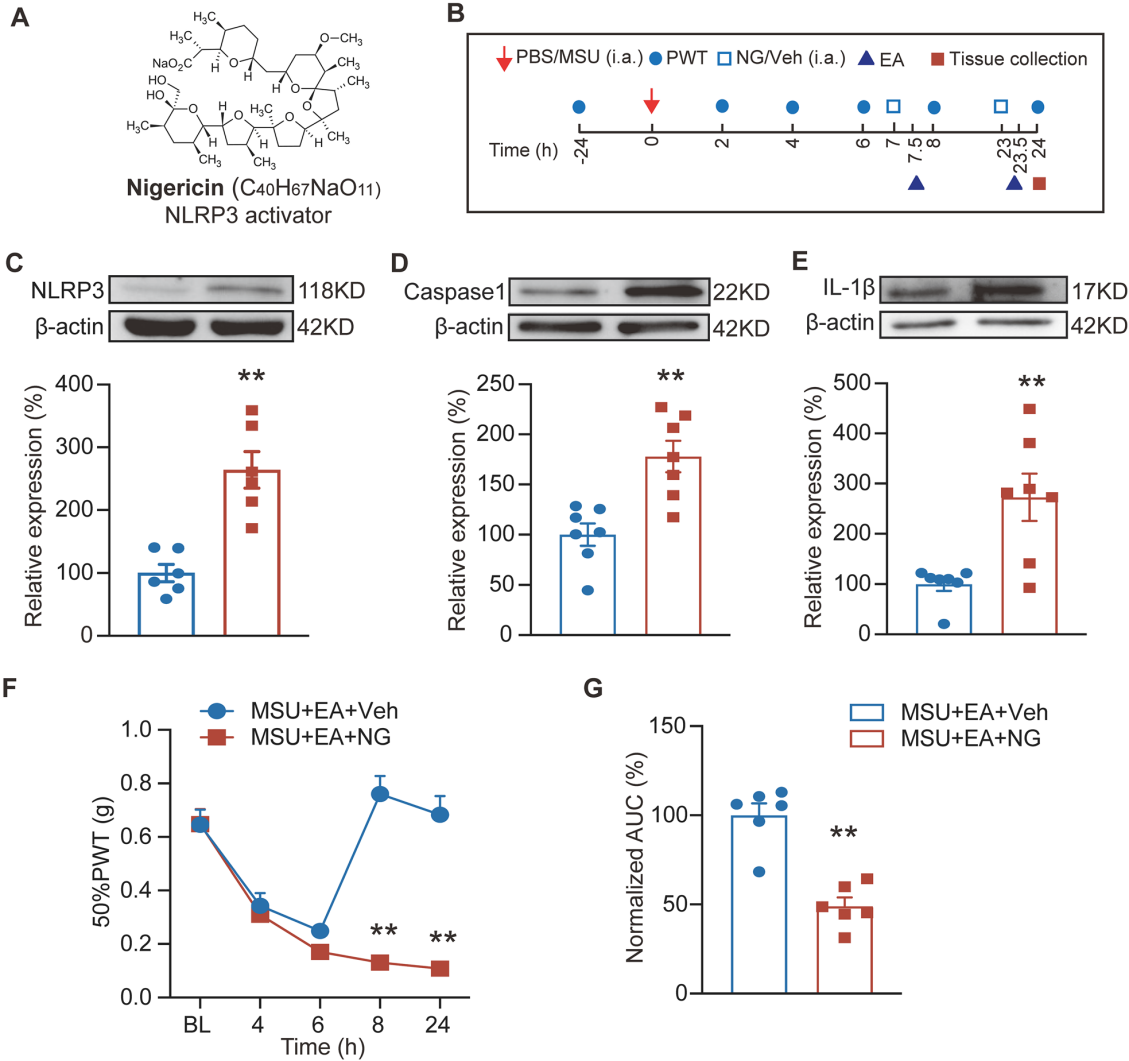
Pharmacological activation of NLRP3 inflammasome in ankle joints reversed EA-induced anti-allodynia in gout model mice. **A** Molecular structure of NLRP3 inflammasome activator nigericin. **B** Schedule illustrating nigericin (NG)/vehicle application, PWT test and tissue collection. (C-E) Western blotting examination of protein expression NLRP3 (**C**), Caspase-1 (**D**) and IL-1β (**E**) in ankle joints of MSU + EA + Veh and MSU + EA + NG groups. **F** Time courses showing 50% PWT changes of ipsilateral hindpaws of MSU + EA + Veh and MSU + EA + NG group mice. **G** Normalized AUC analysis of panel F. n = 5–7 mice/group. *p < 0.05, **p < 0.01 vs. MSU + EA + Veh group

We next investigated whether pharmacologically activating NLRP3 inflammasome could reverse EA-induced anti-allodynia on gout arthritis model mice. To achieve this purpose, nigericin, an activator for NLRP3 [33, 34] (Fig. 5A), was injected into the inflamed ankle joints 30 min before EA intervention (150 μg/mouse, Fig. 5B). Nigericin treatment resulted in obvious NLRP3 inflammasome activation and IL-1β expression increases in ankle joint tissues vs. vehicle-treated group (Fig. 5C–E), showing the effectiveness of nigericin’s action. Furthermore, nigericin treatment reversed EA-induced analgesia vs. vehicle group (Fig. 5F, G). These results indicate that EA’s anti-allodynia on gout model mice can be mimicked by specific pharmacological antagonizing NLRP3 inflammasome and reversed by pharmacological activating NLRP3 inflammasome.

4. EA exerts antioxidative effects to reduce excessive ROS and lipid peroxidation production in ankle joints of gout arthritis model mice.

**Fig. 6:**
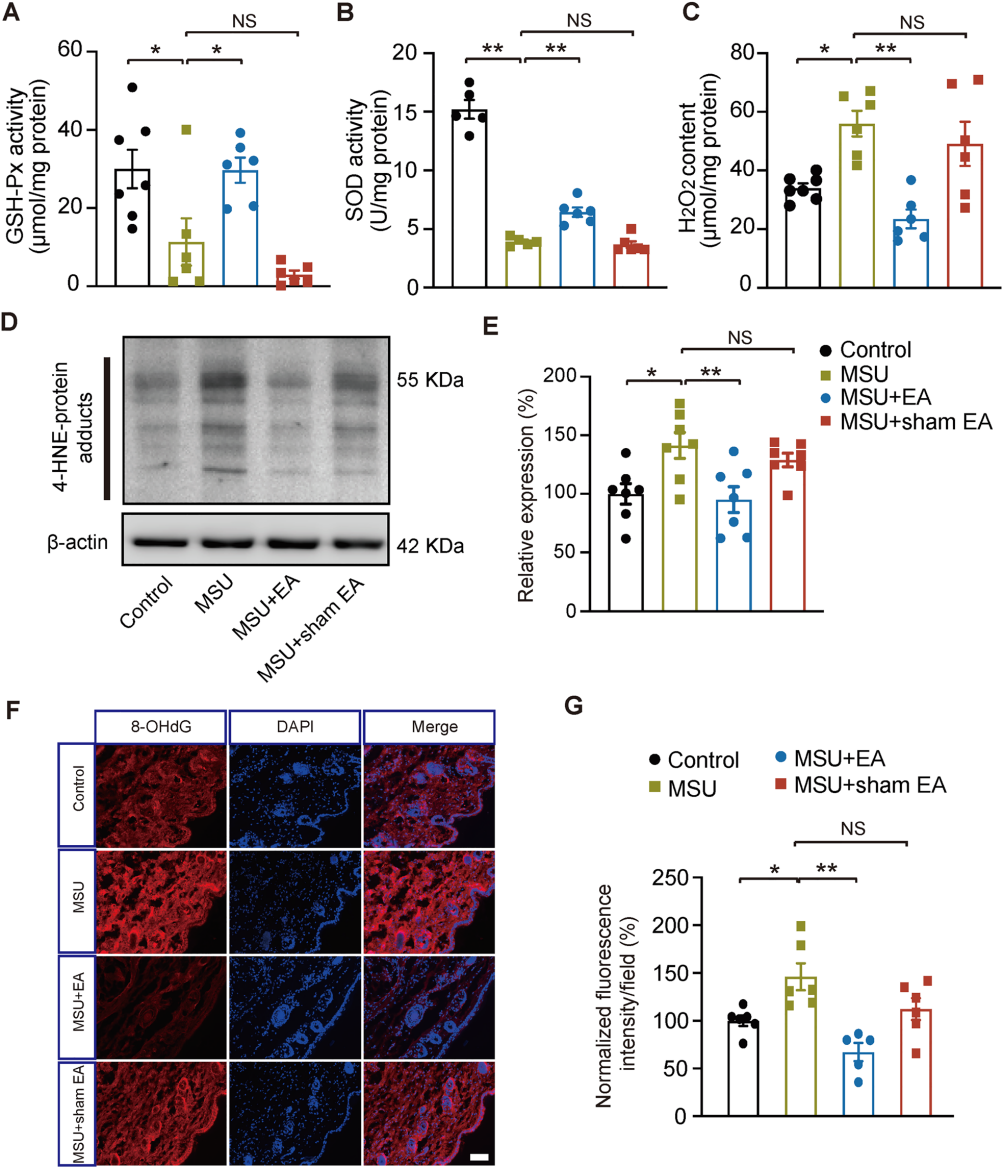
EA intervention attenuates oxidative stress in ankle joint tissues of gout arthritis model mice. **A**–**C** Biochemical assays of oxidative stress markers, including GSH-Px activity, SOD activity and H_2_O_2_ level in control, MSU, MSU + EA and MSU + sham EA groups of mice. **D** Representative gels of Western blotting examining tissue expressions of 4-HNE-protein adducts. **E** Summarized data as in panel D. **F** 8-OHdG immunostaining in periarticular tissues of 4 groups. **G** Summary of 8-OHdG immunostaining in 4 groups. n = 5–8 mice/group. *p < 0.05, **p < 0.01

Our recent studies demonstrate that ROS are excessively produced in ankle joints of gout model mice [11, 12]. The excessive accumulation of ROS contributes to NLRP3 inflammasome activation in inflammation sites [12]. These results indicated that ROS serve as upstream signaling of NLRP3 inflammasome in gout condition [11, 12]. To further learn the mechanisms underlying the interventional effect of EA on NLRP3 inflammasome, we examined whether EA might affect ROS production in inflamed ankle joints of gout model mice. We found that the activity of endogenous antioxidants GSH and SOD was obviously decreased, whereas ROS product H_2_O_2_ content was obviously increased in the joint of gout model group vs. control group (Fig. 6A–C). These observations confirmed the presence of oxidative stress in ankle joints under gout condition. Our results further showed that EA intervention could effectively restore the activities of GSH and SOD and reduced H_2_O_2_ content in model mice (Fig. 6A–C, MSU + EA vs. MSU). But sham EA had no such effect (Fig. 6A–C, MSU + sham EA vs. MSU).

ROS can further attack the cell membrane lipids to generate reactive lipid peroxidation product, e.g. 4-hydroxynonenal (4-HNE), which is capable of activating NLRP3 inflammasome as well [35, 36]. Then we explored the level of 4-HNE by examining the expression of 4-HNE protein adducts through Western blotting. Western blot indicated 4-HNE protein adducts level was obviously increased in the ankle joint tissues of gout model mice. EA intervention significantly reduced 4-HNE protein adducts overexpression in ankle joint tissues of model mice (Fig. 6D, E, MSU + EA vs. MSU). But sham EA had no such effect (Fig. 6D, E, MSU + sham EA vs. MSU). We proceeded to examine oxidative stress-triggered DNA damage using 8-hydroxy-2’-deoxyguanosine (8-OHdG). As shown in Fig. 6F, G, 8-OHdG immunoreactivity was significantly increased in ankle joint sections of gout model mice. EA intervention significantly decreased 8-OHdG immunoreactivity in model mice (MSU + EA vs. MSU). However, sham EA didn’t exert such beneficial effects (MSU + sham EA vs. MSU). These results demonstrate that EA produces anti-oxidative effects and reduces excessive ROS and lipid peroxidation products in ankle joints of gout model mice.

**Fig. 7:**
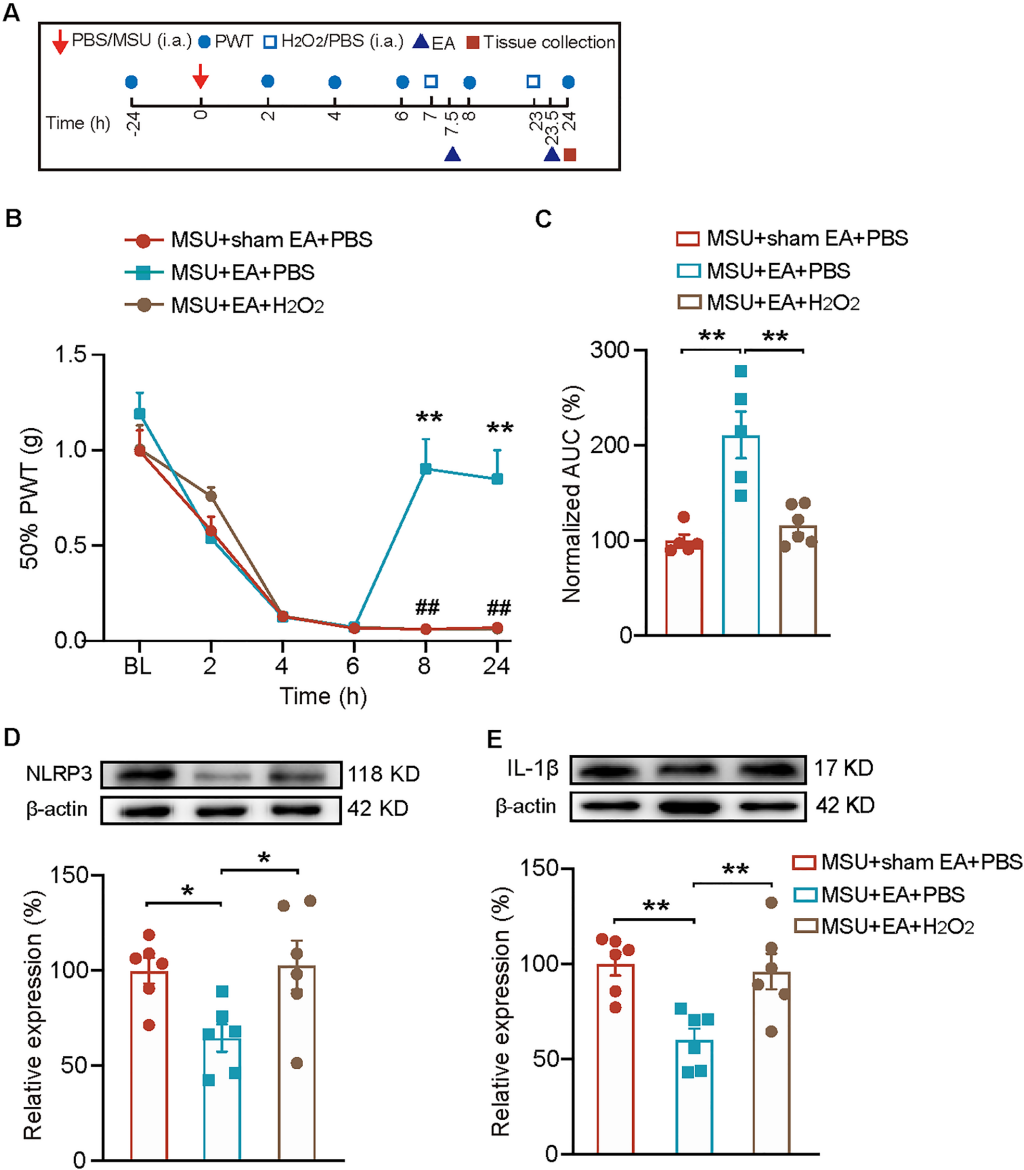
EA-induced antiallodynia and inhibition of NLRP3 inflammasome in model mice are reversed by H_2_O_2_. **A** Schedule depicting the time points for model establishment, H_2_O_2_ and EA application and tissue collection. **B** 50% PWT changes in MSU + EA + H_2_O_2_, MSU + EA + Veh and MSU + ShamEA + Veh groups. **p < 0.01 vs. MSU + sham EA + PBS group, ^##^*p* < 0.01 vs. MSU + EA + PBS. **C** Normalized AUC analysis of curves shown in panel B. (D&E) Western blot of NLRP3 and IL-1β expressions in ankle joint tissues. *p < 0.05, **p < 0.01. n = 6 mice/group

Since EA could target against ROS, we then tested whether applying excessive ROS would reverse EA-induced analgesia by activating NLRP3 inflammasome signaling. We exogenously injected H_2_O_2_ or corresponding vehicle into ankle joints of gout model mice 30 min before EA/sham EA intervention (Fig. 7A). As shown in Fig. 7B, C, exogenously applied H_2_O_2_ significantly reversed EA’s analgesic effect. As a comparison, sham EA produced no analgesic effect (Fig. 7B, C). We continued to explore NLRP3’s expression in the presence of excessive oxidative stress. Figure 7D, E showed that exogenously applied H_2_O_2_ significantly reversed EA’s effect on reducing NLRP3 and IL-1β overexpression in ankle joints of gout model mice. These results indicate that exogenously applied ROS can reverse the anti-allodynia effect and the inhibition of NLRP3 inflammasome by EA in gout model mice.

5. EA intervention attenuates neutrophil infiltrations in ankle joint synovium of model mice.

**Fig. 8:**
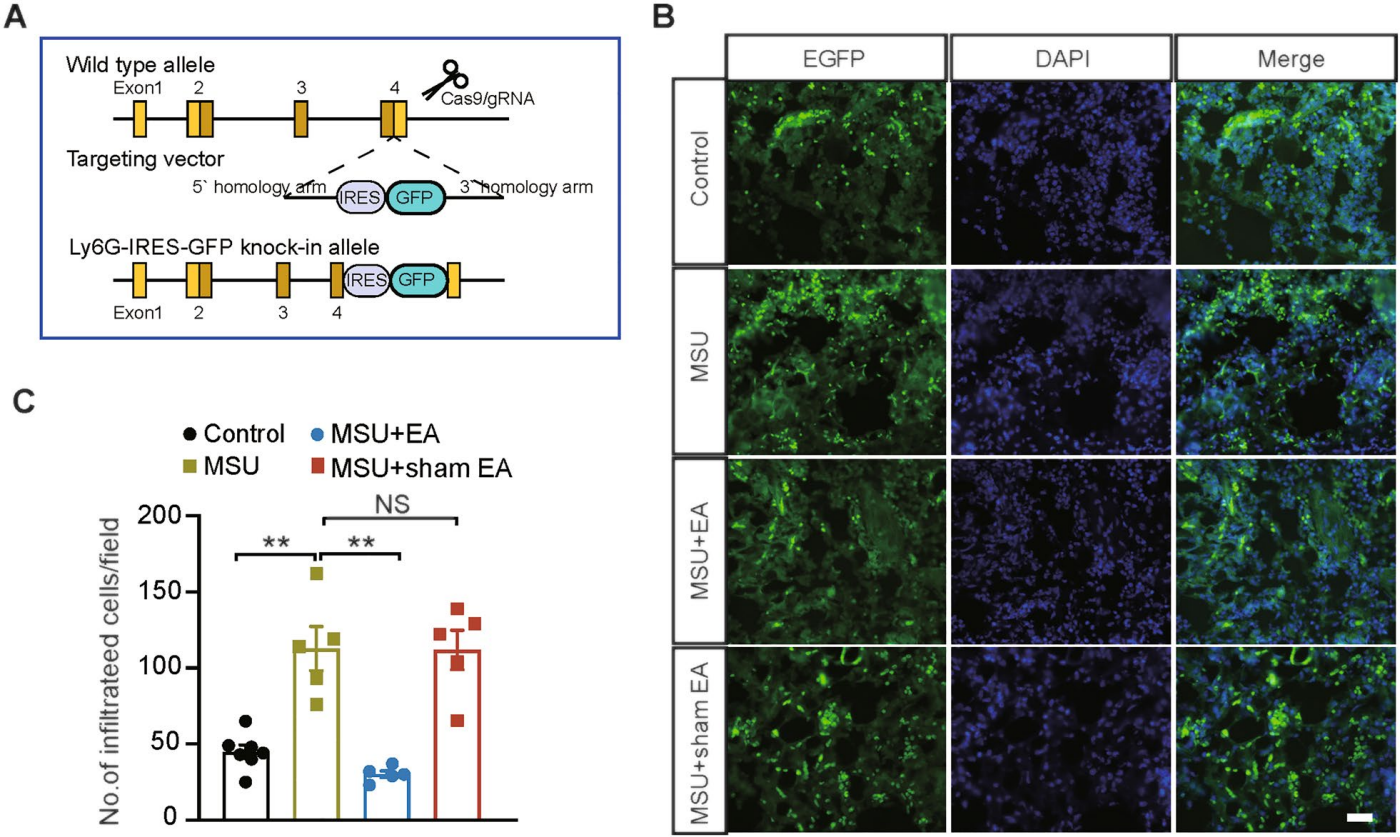
EA intervention attenuates neutrophil infiltrations in synovium of ankle joints of gout arthritis model mice. **A** Cartoon showing the design of Ly6G-IRES-EGFP knock-in mouse line, which enables direct visualization of neutrophils by GFP signal. **B** Representative photos of the fluorescence of Ly6G-EGFP^+^ neutrophils in ankle joint synovarium sections from control, MSU, MSU + EA and MSU + sham EA groups. DAPI was used as a counterstain. Scale bar: 50 μm. **C** Summarized numbers of Ly6g-EGFP^+^ neutrophils/field in each group. **p < 0.01. n = 5–7 mice/group. *NS* no significance

Neutrophil infiltration is considered as a hallmark of acute gout arthritis pathophysiology [32]. Neutrophils initiate respiratory burst, which rapidly releases large amounts of ROS into extracellular space and causes oxidative stress [37]. Our recent work demonstrated that neutrophils constitute a major cellular source for ROS production in inflamed ankle joints of gout model mice [11]. Therefore, we aimed to study whether EA might be able to modulate neutrophil infiltrations to reduce excessive ROS. We generated Ly6g-IRES-GFP knock-in mouse line using Cas9-associated guide RNA (gRNA) technique (Fig. 8A). The mouse line carries the green fluorescence protein (GFP) in Ly6g (a marker for neutrophil) gene allele, which enables the direct monitoring of neutrophils via GFP fluorescence signal. With the aid of this genetically engineered mouse line, we were able to observe that neutrophils exhibited significant infiltrations in the ankle joint synovium of gout model mice (Fig. 8B, C). Furthermore, EA intervention significantly attenuated neutrophil infiltration in ankle joint synovium of gout model mice (Fig. 8B, C, MSU + EA vs. MSU), whereas sham EA did not have such effect (Fig. 8B, C, MSU + sham EA vs. MSU). This result indicates that EA intervention can attenuate excessive neutrophil infiltrations in ankle joints synovium of gout model mice.

6. EA or MCC950 intervention reduced pain-sensing TRPV1 channel up-regulation in dorsal root ganglion (DRG) neurons which innervate the hind limbs of gout arthritis model mice.

**Fig. 9:**
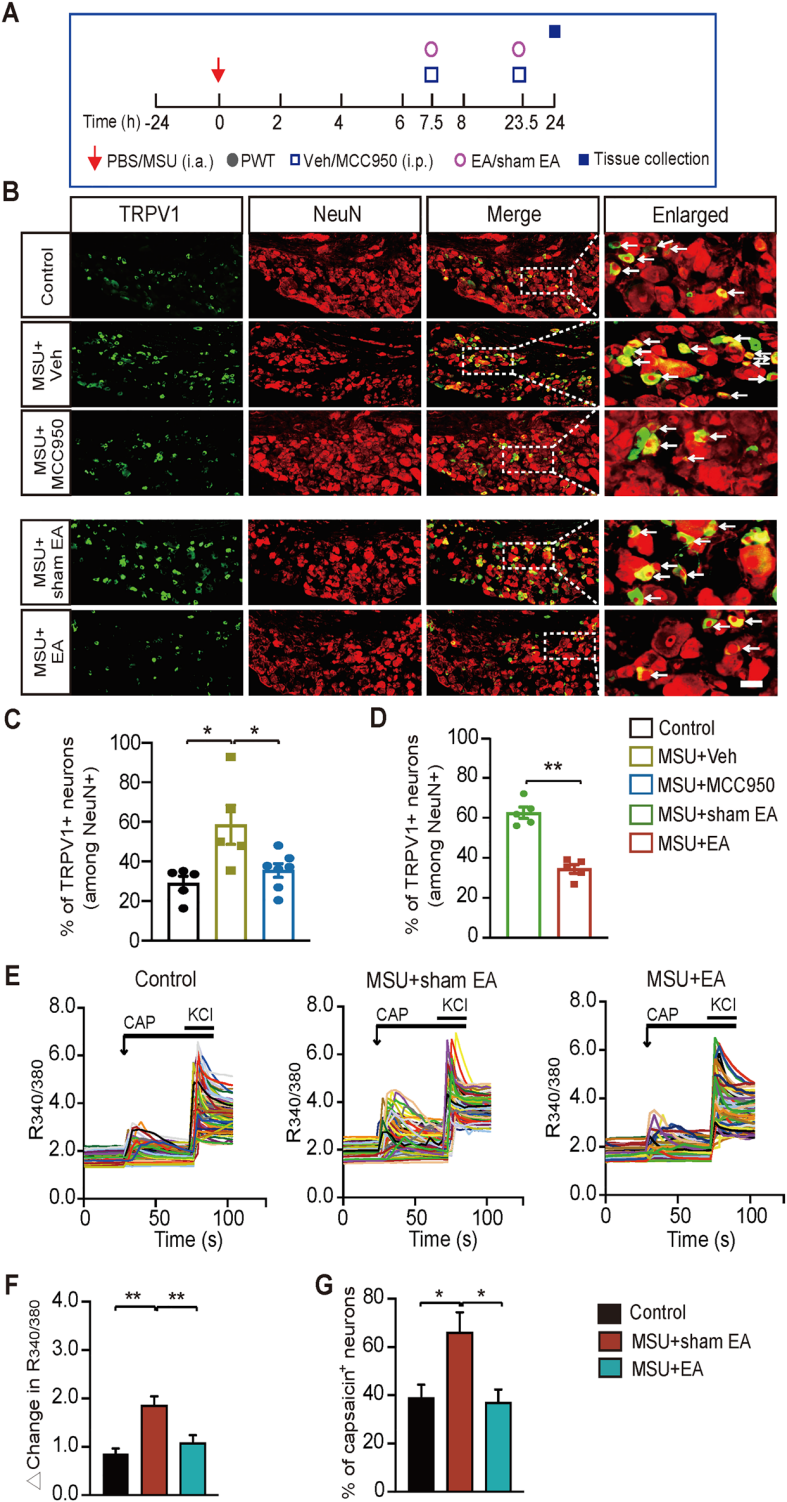
MCC950 or EA intervention reduces pain-sensing TRPV1 channel overexpression in DRG neurons innervating the hindlimbs. **A** Schedule depicting MCC950/vehicle, EA/sham interventions and DRG tissue collection. **B** Representative photos of TRPV1 immunostaining (in green) in DRG neurons across all groups. NeuN staining (in red) was used to identify all DRG neurons. **C**, **D** Summary of the percentage of TRPV1 positively stained (TRPV1^+^) neurons among all DRG neurons in each group. n = 5 mice/group. **E** Representative overlaid Ca^2+^ transients recorded in DRG neurons from control (left panel), MSU + shamEA (middle panel) and MSU + EA (right panel) group of mice. Capsaicin (300 nM) was applied to activate TRPV1 channels. KCl (40 mM) was perfused to determine all alive neurons. 90 cells were included in each panel. **F** Summarized data for delta increase in peak R_340/380_ caused by capsaicin of 3 groups. n = 5–6 tests/group. **G** Summarized data for the percentage of capsaicin positively responding (capsaicin^+^) neurons of 3 groups. > 200 neurons were included in each group. *p < 0.05, **p < 0.01

The pain-sensing TRPV1 channel makes important contributions to mechanical pain and joint inflammation in gout condition [38, 39]. Our recent study found TRPV1 expression is up-regulated in DRG neurons innervating ankle joints of gout arthritis model mice [12]. IL-1β can promote TRPV1 channel overexpression in sensory neurons and contribute to peripheral sensitization [40]. We then asked whether TRPV1 channel expression is up-regulated via NLRP3 inflammasome dependent mechanism and whether EA intervention would attenuate TRPV1 channel up-regulation in DRG neurons of gout arthritis model mice. We performed immunostaining on ipsilateral L3-L5 DRG neurons which innervate the hind paw at 24 h time point (Fig. 9A). The results indicated a higher percentage of TRPV1 positively stained (TRPV1^+^) DRG neurons among all DRG neurons in MSU group, a result consistent with our recent study (Fig. 9B, C) [12]. Blocking NLRP3 inflammasome via MCC950 obviously attenuated TRPV1 channel overexpression in DRG neurons (Fig. 9B, C), indicating NLRP3 inflammasome is involved in TRPV1 channel overexpression in DRG neurons of gout arthritis model mice. EA intervention effectively decreased TRPV1 channel overexpression compared with sham EA in gout model mice (Fig. 9B, D, MSU + EA vs. MSU + sham EA). This result indicates that EA intervention attenuates TRPV1 channel overexpression in DRG neurons, which is possibly mediated via attenuation of NLRP3 inflammasome activation.

We further monitored the functional activity of TRPV1 channel in acutely dissociated DRG neurons via live cell ratiometric Ca^2+^ imaging. As shown in Fig. 9E, F, the TRPV1 specific agonist capsaicin (300 nM) produced stronger Ca^2+^ transients in DRG neurons isolated from MSU + sham EA group than control group. EA intervention reduced the exaggerated Ca^2+^ transients triggered by capsaicin (Fig. 9E, F). The percentage of capsaicin reactive (capsaicin^+^) cells in MSU + sham EA group is significantly more than control group. The increased capsaicin responsiveness was also significantly reduced by EA (Fig. 9E, G). These results indicate that EA intervention can reduce the enhanced TRPV1 channel activity in DRG neurons in gout condition.

## Discussion

Gout arthritis is triggered by excessive MSU accumulations in joints. MSU induces NLRP3 inflammasome activations. Peripheral IL1β is involved in mediating varies pain conditions by directly activating the nociceptors or by producing peripheral sensitization [41]. Pharmacological blockage of IL-1β signaling shows profound effectiveness for attenuating gout pain [42]. Furthermore, NLRP3 knockout mice showed reduced mechanical allodynia induced by MSU [32]. These findings in all proposed a pivotal role of NLRP3 signaling in mediating gout pain [10]. EA was reported to be able to reduce the activation of NLRP3 inflammasome in peripheral tissues and spinal cord in some specific pain model animals [43, 44]. But it is still elusive if NLRP3 inflammasome indeed contributes to the pain mechanisms in those specific pain conditions. Unfortunately, the causal relationship between EA-mediated NLRP3 inflammasome inhibition and EA-mediated analgesia is still not fully elucidated. Recently, our group identified that NLRP3 inflammasome is activated in neurons of spinal cord dorsal horn (SCDH) and contributes to mechanical allodynia of a rat model of CRPS-I [45, 46]. We further showed that EA attenuates mechanical pain responses by reducing NLRP3 inflammasome activation in spinal neurons [45]. But it remains unknown whether EA could exert similar effects on NLRP3 inflammasome to attenuate gout arthritis pain in peripheral joint tissues. Here, our results indicated that EA remarkably decreased NLRP3 inflammasome components’ overexpression, including NLRP3, Caspase-1 and IL-1β of gout model animals. Specific blocking NLRP3 inflammasome mimics the effect of EA, whereas activating NLPR3 reverses it. These results indicate that EA attenuates gout arthritis pain via mechanisms possibly involving interventional effects on NLRP3 inflammasome.

Previous work showed that ROS activate NLRP3 inflammasome [47]. Moreover, MSU activates NLRP3 inflammasome through ROS-dependent mechanism [14]. Recent work indicates that large amounts of ROS products are overproduced from local ankle joints of model mice and blocking ROS by antioxidants or natural anti-oxidative products is effective in reducing NLRP3 inflammasome activation in gout model mice [12, 48], suggesting ROS is critical for NLRP3 inflammasome activation during gout. We then aimed to explore if EA might affect ROS production in gout condition. Our data showed that EA obviously decreased H_2_O_2_ and 4-HNE levels in ankle joints of model mice. EA further reduced oxidative stress-induced tissue damage. More importantly, EA-induced anti-allodynia was reversed by exogenously applying H_2_O_2_. These results in together suggest that EA produces anti-oxidative effect on gout model mice, resulting in less NLRP3 inflammasome activation.

We further explored how EA might exert anti-oxidative effects in gout condition. Previous study indicated that neutrophils exert respiratory burst that can rapidly release large amounts of ROS to increase oxidative stress [37]. Recent work further indicated that blocking neutrophil infiltrations significantly reduced ROS production as well as gout pain in the inflamed ankle joint of gout model mice, indicating that neutrophils make important contributions to ROS overproduction and joint pain in gout condition [11]. Recent work indicated that EA can attenuate neutrophil accumulation in the spleen of a chronic inflammatory pain model mice [44]. Following these prior studies, we examined whether EA could affect neutrophil infiltration in local inflamed ankle joints of gout model mice. We found that EA intervention could significantly attenuate neutrophil infiltration in local ankle synovium of gout model mice, which in turn may result in less ROS production and pain alleviation. But it should be noted that, in addition to the possible mechanism mentioned above, EA may also affect endogenous antioxidant signaling, e.g. the nuclear factor erythroid 2-related factor 2 (Nrf2), to exert antioxidative effect. Nrf2 antioxidant signaling represents a crucial endogenous mechanism which serves to control excessive oxidative stress by enhancing the activation of an array of antioxidant genes’ expression [49]. Our recent work showed that EA could reduce oxidative stress injuries in an animal model of CRPS-I. This work further showed EA’s mechanism of action was exerted via enhancing Nrf2-mediated antioxidative mechanisms to relieve pain and inflammation of the affected hind paw [45]. Therefore, it is also likely that EA may enhance Nrf2-mediated endogenous antioxidant signaling in inflamed ankle joints to counteract oxidative stress in gout condition. Therefore, further studies will be needed to examine the possible contributions of Nrf2 in EA-mediated antioxidative effect in gout condition.

TRPV1 channel has been shown to contribute to gout pain and inflammation. Previous work indicated that pharmacological blocking TRPV1 is effective in reducing gout pain, joint inflammation and inflammatory cell infiltration [38, 39]. TRPV1 expression in DRG neurons is significantly up-regulated in gout model mice [12]. Here, we further found TRPV1 channel functional activities were up-regulated in sensory neurons of gout model mice. Previous work also indicated that TRPV1 expression can be promoted by IL-1β and many other pro-inflammatory cytokines, which constitutes a critical mechanism contributing to peripheral sensitization [23, 40]. However, the mechanisms underlying TRPV1 up-regulation in peripheral sensory neurons during gout are still not fully understood. Here, our results showed that pharmacological blockage of NLRP3 inflammasome remarkably attenuated TRPV1 overexpression in sensory neurons of gout mice, indicating NLRP3 inflammasome contributes to TRPV1 overexpression in gout condition. Our data further indicated that EA mimics the effects of MCC950 to reduce NLRP3 inflammasome activation and reduce TRPV1 overexpression. EA’s effect on functional TRPV1 channel expression is further confirmed by live cell Ca^2+^ imaging experiments in this study. Therefore, our work suggests that EA may attenuate TRPV1 overexpression in sensory neurons of gout model mice through mechanisms involving the reduction of NLRP3 inflammasome activation.

In addition to TRPV1, some other TRP channels have also been reported to contribute to gout arthritis pain and inflammation. It is known that ROS or the related lipid peroxidation products produced from inflamed tissues can activate pain-sensing TRPA1 channel expressed on sensory nerve endings and produce pain signal [50, 51]. Genetic ablation or pharmacological inhibition of TRPA1 reduced mechanical hypersensitivity of gout model mice, suggesting ROS may act upon TRPA1 channel to produce gout arthritis pain [13, 48]. In addition, TRPV4 has recently been reported to contribute to NLRP3 inflammasome activation in macrophages in gout arthritis. Specific deleting TRPV4 expression in macrophages results in attenuated pain response and inflammation in gout model mice [52]. Therefore, it will be tempting to explore EA’s effect on TRPA1- or TRPV4 channel activity and related cellular signaling in gout arthritis in future studies. These studies in together may help to unravel EA’s analgesic effect on gout arthritis pain to further extent.

## Conclusion

Our work demonstrates that EA intervention alleviates pain as well as the related gait impairment in a mouse gout arthritis model. EA’s effect may involve the suppression of ROS-mediated NLRP3 inflammasome activation in inflamed joint tissues and TRPV1 channel upregulation in the sensory neurons. This work further supports EA to be used as an alternative therapeutic option for gout pain management.

## Supplementary Information

**Additional file 1: Figure S1.** Spatiotemporal characterization of hindpaw area changes of gout arthritis model mice by EA intervention via gait analysis

## Data Availability

The original contributions presented in the study are included in the article/supplementary material, further inquiries can be directed to the corresponding author/s.

## Abbreviations

MSU: Monosodium urate
PWT: Paw withdrawal threshold
EA: Electroacupuncture
DRG: Dorsal root ganglion
SOD: Superoxide dismutase
GSH: Glutathione
ROS: Reactive oxygen species
PWL: Paw withdrawal latency
