# Supplementary material for: Electroacupuncture improves gout arthritis pain via attenuating ROS-mediated NLRP3 inflammasome overactivation
Source: Chin Med. 2023 Jul 18;18:86. doi: 10.1186/s13020-023-00800-1 (PMC10355064; doi:10.1186/s13020-023-00800-1)
Supplement: Supplementary file 1 — Additional file 1: Figure S1. Spatiotemporal characterization of hindpaw area changes of gout arthritis model mice by EA intervention via gait analysis [file 13020_2023_800_MOESM1_ESM.docx]

**Figure S1**

**

**Fig. S1 Spatiotemporal characterization of hindpaw area changes of gout arthritis model mice by EA intervention via gait analysis.** (A) Representative data showing dynamic changes of ensemble hindpaw area of the righ vs. left hindpaw from control, MSU, MSU+EA and MSU+sham EA group mouse at 8 and 24 h after model establishment, respectively. (B&C) Summarized and overlaid dynamic changes in ensemble hindpaw area obtained from all 4 groups at 8 h (B) and 24 h (C) time point after model establishemnt. n = 6 mice/group. **p*<0.05, ***p*<0.01. NS: no significance. Two-way ANOVA with Tukey’s post-hoc test was used.
